# Curcumin Attenuates β-catenin Signaling in Prostate Cancer Cells through Activation of Protein Kinase D1

**DOI:** 10.1371/journal.pone.0035368

**Published:** 2012-04-16

**Authors:** Vasudha Sundram, Subhash C. Chauhan, Mara Ebeling, Meena Jaggi

**Affiliations:** 1 Cancer Biology Research Center, Sanford Research/USD, Sioux Falls, South Dakota, United States of America; 2 Department of OB/GYN and Basic Biomedical Science Division, Sanford School of Medicine, The University of South Dakota, Sioux Falls, South Dakota, United States of America; National Cancer Center, Japan

## Abstract

Prostate cancer is the most commonly diagnosed cancer affecting 1 in 6 males in the US. Understanding the molecular basis of prostate cancer progression can serve as a tool for early diagnosis and development of novel treatment strategies for this disease. Protein Kinase D1 (PKD1) is a multifunctional kinase that is highly expressed in normal prostate. The decreased expression of PKD1 has been associated with the progression of prostate cancer. Therefore, synthetic or natural products that regulate this signaling pathway can serve as novel therapeutic modalities for prostate cancer prevention and treatment. Curcumin, the active ingredient of turmeric, has shown anti-cancer properties via modulation of a number of different molecular pathways. Herein, we have demonstrated that curcumin activates PKD1, resulting in changes in β-catenin signaling by inhibiting nuclear β-catenin transcription activity and enhancing the levels of membrane β-catenin in prostate cancer cells. Modulation of these cellular events by curcumin correlated with decreased cell proliferation, colony formation and cell motility and enhanced cell-cell aggregation in prostate cancer cells. In addition, we have also revealed that inhibition of cell motility by curcumin is mediated by decreasing the levels of active cofilin, a downstream target of PKD1. The potent anti-cancer effects of curcumin *in vitro* were also reflected in a prostate cancer xenograft mouse model. The *in vivo* inhibition of tumor growth also correlated with enhanced membrane localization of β-catenin. Overall, our findings herein have revealed a novel molecular mechanism of curcumin action via the activation of PKD1 in prostate cancer cells.

## Introduction

Prostate cancer is the second leading cause of death and the most commonly diagnosed cancer in males in the US [1]. The risk for prostate cancer increases exponentially after the age of 50. Hence, prostate cancer is positioned to become a greater challenge in the coming years due to an overall increase in longevity. While the etiology of prostate cancer is not well understood, both genetic and environmental factors seem to play important roles in the development of the disease. A common first-line strategy for treatment of prostate cancer includes surgical or pharmacological castration through androgen ablation therapy. While androgen ablation therapy is effective during initial stages of the disease, the cancer quickly progresses to an androgen independent stage for which no known effective therapy is currently available. Therefore, understanding the molecular basis of the disease is highly desirable for developing newer strategies for prevention and treatment of prostate cancer.

Protein Kinase D1 (PKD1) is an evolutionarily conserved, ubiquitously expressed serine-threonine kinase that plays a central role in regulating a variety of cellular functions including cell survival, proliferation, motility and invasion [2]–[8]. The *PKD1* gene is expressed in many organs with the highest expression documented in the prostate and testis germ cells [3], [9], [10]. PKD1 exhibits a combination of structural and functional features of both the PKC family (diacyl glycerol and phorbol ester binding structural domains) and the CaMK family (structural homology of kinase domain and substrate and inhibitor specificity). Therefore, it is uniquely positioned within the signal transduction cascade for integrating signaling information from external stimuli and converts them into intracellular response by modulating diverse downstream pathways [2]. Thus, the deregulation of PKD1 affects multiple signaling pathways, resulting in chronic diseases like cancer [2]. Previous work from our laboratory has implicated a critical role for PKD1 in prostate cancer [11]. Our work has revealed the ability of PKD1 to inhibit the functions of β-catenin in prostate cancer [12]. In addition, PKD1 has been shown to interact with and modulate the functions of E-cadherin, androgen receptor and MAPKinase signaling pathways [13]–[18]. PKD1 also inhibits cell motility by directly interacting with and modulating the functions of a number of proteins involved in actin remodeling, including sling shot phosphatase (SSH1L) and cortactin [19]–[23]. Furthermore, PKD1 is known to be involved in inhibiting invasion, metastasis and epithelial-mesenchymal transition (EMT) of cancer cells by regulating the expression of matrix metalloproteinases (MMPs) [24], [25] and the functions of snail transcription factor [26], respectively. Therefore, molecules that modulate PKD1 expression or activity may play an important role in the prevention and or treatment of prostate cancer.

**Figure 1:**
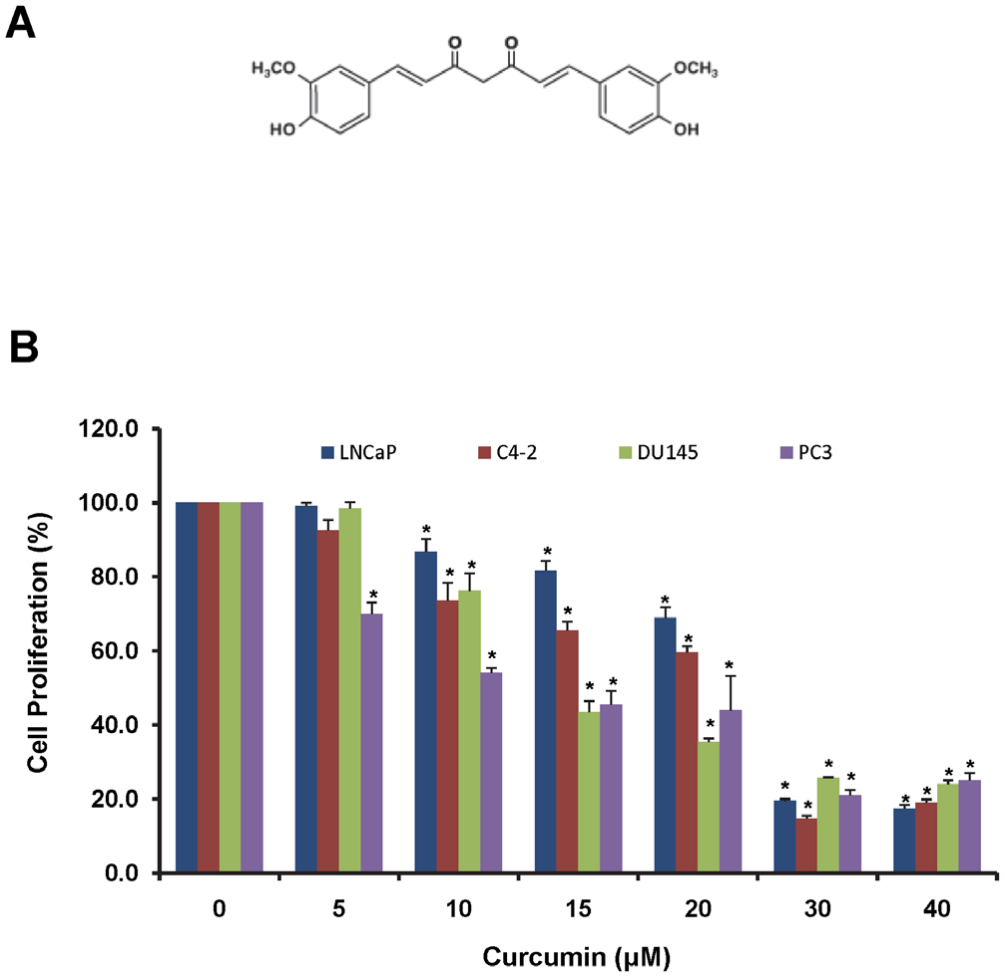
Curcumin inhibits prostate cancer cell proliferation. A). Chemical structure of curcumin. B). Effect of curcumin on proliferation of various prostate cancer cell lines. LNCaP, C4-2, DU145 and PC3 cell were treated with curcumin or vehicle control DMSO for 48 h and cell proliferation was determined using MTS assay. The percent cell proliferation was calculated by normalizing the proliferation of curcumin treated cells with proliferation of control treated cells. Concentration dependent inhibition in cell proliferation was observed with curcumin treatment. Mean ± SE; n = 3; *p<0.05.

Curcumin (Figure 1A), the active ingredient of turmeric, is a non-toxic, diferuloyl methane compound that has potent anti-proliferative, anti-inflammatory and anti-oxidative properties [27], [28]. Both *in vivo* and *in vitro* studies have demonstrated the ability of curcumin to effectively inhibit cancer growth [29]–[31]. This potent anti-cancer property of curcumin is related to its ability to simultaneously modulate the functions of a number of different molecular pathways including MAPK, EGFR and NFκB pathways [32]. In addition, curcumin also regulates the nuclear β-catenin/T cell factor (TCF) transcriptional activity. However, the precise molecular mechanisms of curcumin mediated suppression of β-catenin transcriptional activity are not fully understood.

In the present study, we have revealed the effect of curcumin on PKD1 activation. Curcumin mediated PKD1 activation suppressed nuclear β-catenin/TCF transcription activity and inhibited the growth of prostate cancer in cell line and xenograft animal model. Activation of PKD1 also enhanced cell-cell aggregation and inhibited cell motility functions *via* enrichment of membrane β-catenin and the inhibition of cofilin activity. Overall, we have revealed a novel molecular signaling pathway regulated by curcumin to attenuate prostate cancer growth.

## Results

### Curcumin inhibits prostate cancer cell proliferation

Deregulated cell proliferation is a hallmark of cancer cells. In order to determine the anti-proliferative activity of curcumin (Figure 1A), a panel of prostate cancer cell lines (early passage androgen sensitive LNCaP, androgen-independent C4-2, PC3 and DU145) were treated with varying concentrations of curcumin for 48 h and assessed for cell proliferation. Curcumin treatment exhibited dose-dependent inhibition of cell proliferation in all the different prostate cancer cells (Figure 1B). The paired prostate cancer cell line system LNCaP (androgen sensitive) and C4-2 cells (LNCaP derived, androgen-independent) were used for further studies.

### Curcumin activates PKD1

**Figure 2:**
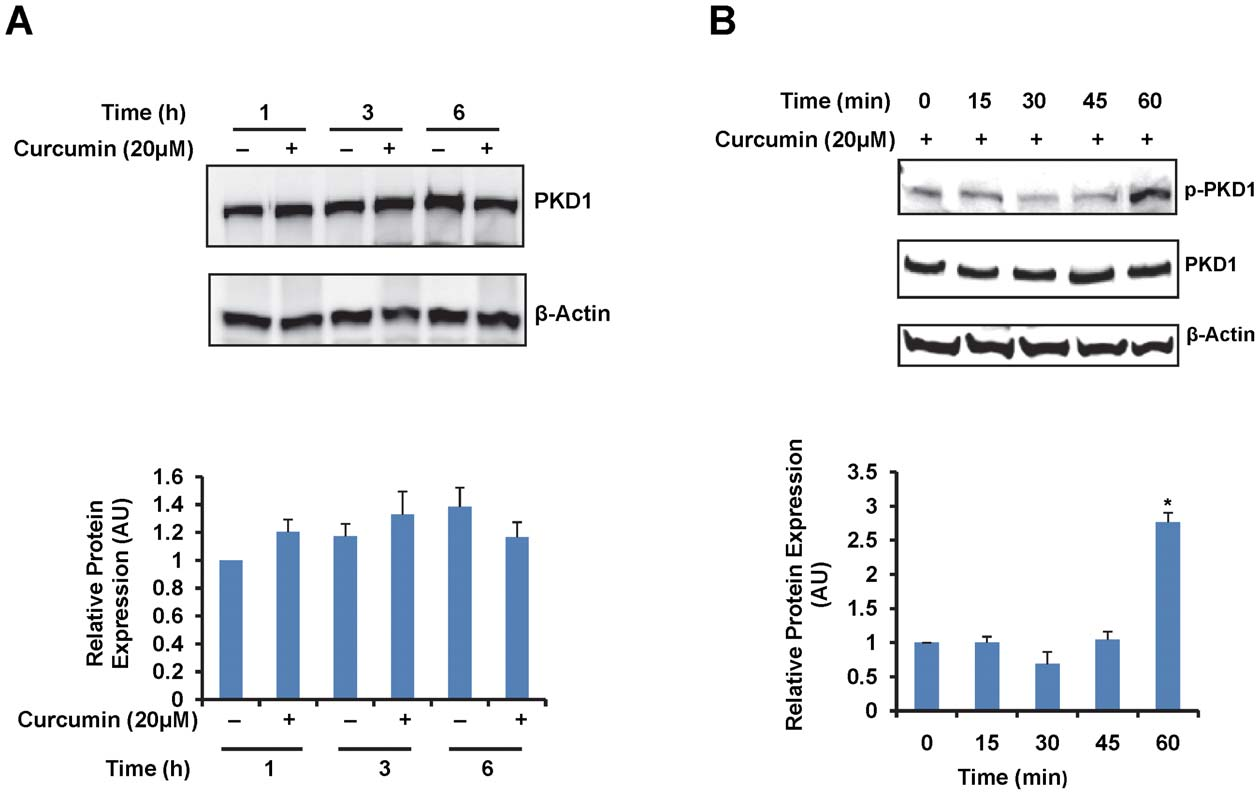
Curcumin activates PKD1. A). Effect of curcumin on PKD1 levels. C4-2 cells were treated with 20 µM curcumin. At varying time points, the cells were harvested, and the lysates were resolved on SDS-PAGE, transferred onto a PVDF membrane and probed for total PKD1. β-actin was used as an internal loading control. The band intensities were densitometrically analyzed, normalized to β-actin levels and graphed. Curcumin treatment resulted in no marked change in PKD1 expression at 1, 3 and 6 h. B). Effect of curcumin on activation of PKD1. For determining the expression of activated/phosphorylated PKD1, blots were probed with phospho PKD1, total PKD1 and β-actin antibodies. The pPKD1 band intensity was normalized to total PKD1 levels and graphed. Curcumin treatment induced PKD1 activation/phosphorylation by 1 h, while no apparent changes were observed in the expression of total PKD1. Representative blots of three experiments are shown in the figure. AU- arbitrary units.

PKD1 is necessary for normal physiology of the prostate cells and its down regulation is associated with the progression of prostate cancer. Therefore, non-toxic, natural compounds that upregulate the expression or activity of PKD1 may help in the prevention and or treatment of prostate cancer. In order to determine if curcumin can modulate the expression or activity of PKD1, androgen-independent prostate cancer C4-2 cells were treated with curcumin for varying time points, and the expression of total PKD1 (Figure 2A) and active PKD1 (Figure 2B) was determined using PKD1 and phospho PKD1 antibody antibodies. While no marked change in the total PKD1 level was detected following curcumin treatment (Figure 2A), curcumin treatment significantly enhanced the expression of activated PKD1 within 1 h of treatment (Figure 2B and Figure S1A). Similar results were also obtained in C4-2 cells overexpressing PKD1 (C4-2-PKD1) (Figure S1B) and in LNCaP cells (Figure S1C). These data suggest that curcumin treatment can efficiently induce the activation of PKD1 in prostate cancer cells.

### Curcumin treatment enriches β–catenin localization at the cell membrane

**Figure 3:**
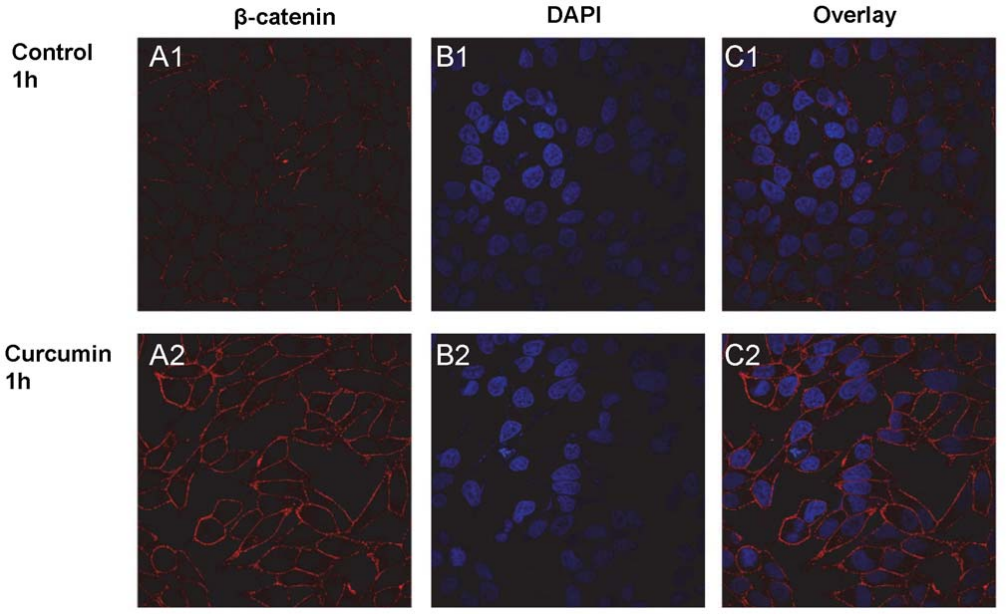
Curcumin treatment enhances membrane β-catenin. C4-2 cells were cultured on glass coverslips overnight in 12 well plates. The cells were treated with DMSO (upper panel) or curcumin (20 µM) (lower panel) for 1 h, washed, fixed and immunostained for β-catenin (red) and counter-stained with DAPI (blue). Higher β–catenin staining was observed on the cell surface at 1 h of curcumin treatment, compared to DMSO control treated cells. Original Magnifications 600×.

β-catenin is an important cellular protein that is phosphorylated by PKD1. PKD1 has also been shown to increase the levels of membrane β-catenin and cell-cell interaction in prostate cancer cells [12]. To determine the effect of curcumin mediated activation of PKD1 on membrane β-catenin localization, curcumin treated C4-2 cells were immunostained for β-catenin and processed for confocal microscopy (Figure 3). As shown in Figure 3, curcumin treatment enriched membrane β-catenin localization in C4-2 cells within 1 h of treatment compared to vehicle control treated cells. Similar results were also observed in LNCaP cells (Figure S2).

### Curcumin mediated enhancement of membrane β-catenin is inhibited by PKD1 siRNA

**Figure 4:**
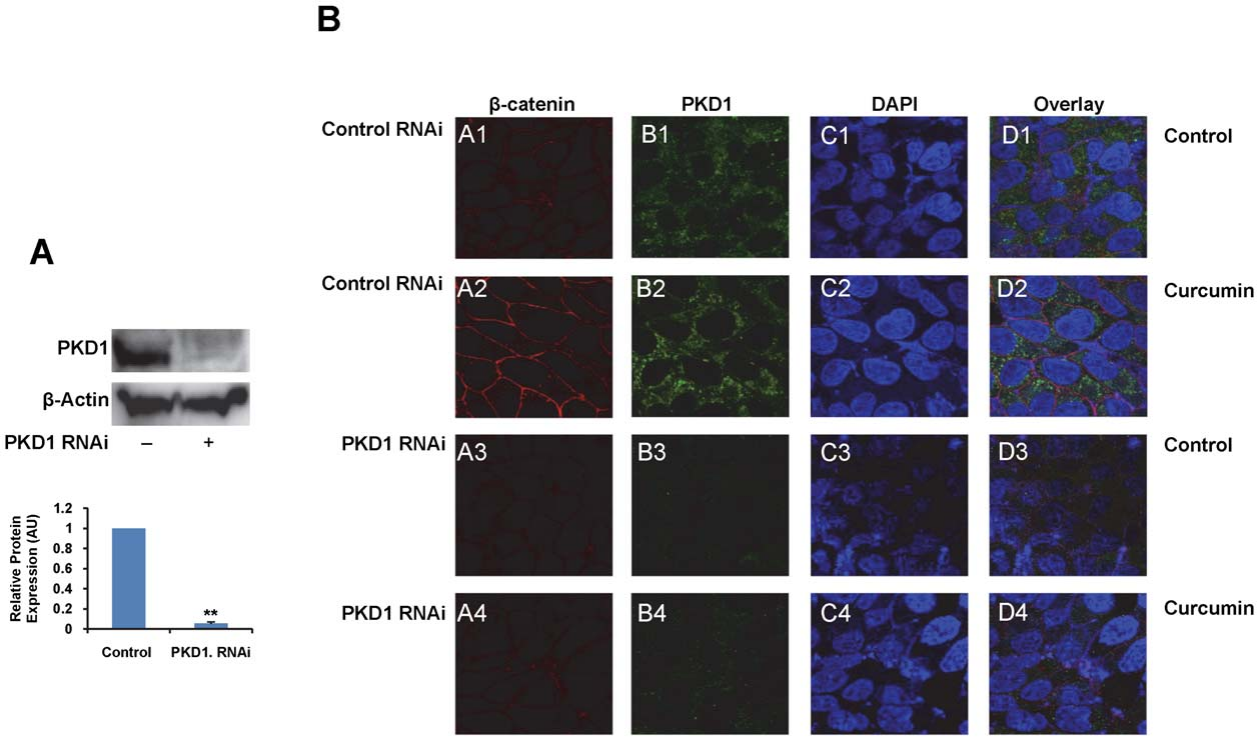
PKD1 is required for curcumin induced enrichment of β-catenin on the membrane. A). Silencing of PKD1 by PKD1 specific siRNA. C4-2 cells were transfected for 48 h with 25 nM control siRNA or PKD1 siRNA, lysed and immunoblotted for PKD1 and β-actin using specific antibodies. Quantitation of protein band intensities was performed by densitometric analysis. The PKD1 levels was normalized to β-actin levels and graphed. AU- arbitrary units. Immunoblotting shows over 95% suppression of PKD1 expression on transfection with PKD1 specific siRNA (lane 2) compared to control siRNA-transfected cells (lane 1). B). Suppression of PKD1 inhibits enrichment of membrane β-catenin levels. C4-2 cells were cultured on coverslips overnight. The cells were first transfected with either control siRNA (A1–D1; A2–D2) or PKD1 silencing siRNA (A3–D3; A4–D4) for 24 h, followed by treatment with vehicle control (DMSO) (A1–D1; A3–D3) or curcumin (20 µM) (A2–D2; A4–D4) for 1 h. The cells were immunostained for β-catenin (red) or PKD1 (green) and the nucleus was counter stained with DAPI (blue). Higher β–catenin staining was observed on the cell surface of control siRNA cells at 1 h of curcumin treatment (A2) compared to vehicle treatment (A1). However, siRNA mediated silencing of PKD1 (B3, B4) inhibited curcumin mediated enrichment of membrane β–catenin staining on the cell surface (A4 vs A3 and A2). Original Magnifications 600× with 2× zoom.

PKD1 has previously been shown to influence the subcellular localization of β-catenin [12]. Therefore, we sought to investigate the role of PKD1 in curcumin mediated enrichment of membrane β-catenin. For this purpose, we used PKD1 specific siRNA to silence PKD1 in C4-2 cells. PKD1 siRNA effectively silenced PKD1 expression (over 95%) in C4-2 cells compared to scrambled (non-targeted) siRNA (Figure 4A). After inhibition of PKD1 expression, C4-2 cells were treated with curcumin and processed for confocal microscopy to determine β-catenin and PKD1 expression and localization (Figure 4B). In scrambled siRNA transfected cells, curcumin treatment efficiently enhanced β-catenin localization on the cell membrane (Figure 4B, A2–D2) compared to control cells (Figure 4B, A1–D1). However, in cells transfected with PKD1 silencing siRNA, curcumin treatment failed to enrich β-catenin on the membrane in C4-2 cells (Figure 4B, A4–D4). These data suggest that PKD1 plays a role in curcumin mediated enrichment of membrane β-catenin in prostate cancer cells.

### Curcumin attenuates nuclear β-catenin signaling

**Figure 5:**
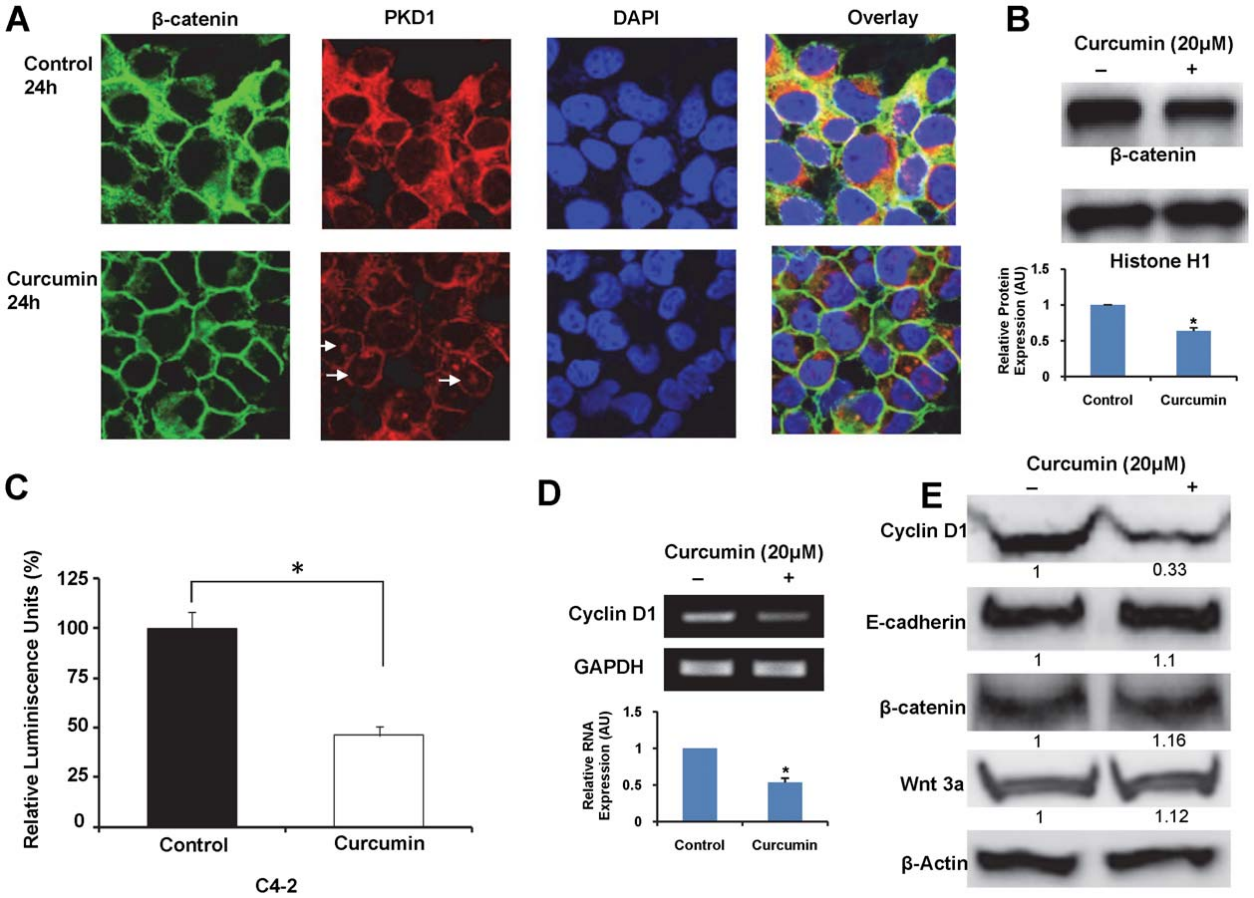
Curcumin inhibits β-catenin transcription activity in prostate cancer cells. A) Effect of curcumin treatment on the cellular localization of β-catenin and PKD1. C4-2 cells treated with curcumin (20 µM) or DMSO for 24 h were immunostained for β-catenin (green) or PKD1 (red) and counter-stained with DAPI (blue). Curcumin treated cells showed lower cytoplasmic and higher membrane β-catenin staining compared to control cells. In addition, while PKD1 was predominantly localized in the cytoplasm in control cells, curcumin treated cells exhibited staining primarily on the cell membrane and in the nucleus (white arrows), with faint cytoplasmic staining. Original Magnifications 600× with 2× zoom. B) Effect of curcumin on nuclear β-catenin levels. Nuclear proteins isolated from C4-2 cells treated either with curcumin (20 µM) or DMSO were resolved on PVDF membrane and processed for immunoblotting using β-catenin antibody. Histone H1 protein was used as loading control. Densitometric quantitation of β-catenin band intensities, normalized to Histone H1 levels is shown in graph. Curcumin treatment markedly decreased the levels of nuclear β-catenin compared to vehicle treated cells. AU- arbitrary units. C) Effect of curcumin on β-catenin transcription activity in C4-2 prostate cancer cells. The β-catenin transcription activity was measured by transiently transfecting the cells with TCF luciferase reporter construct containing either TCF promoter binding sites (pTOP-FLASH) or mutant TCF promoter binding sites (pFOP-FLASH) along with internal control plasmid containing *Renilla* luciferase gene (pRL-TK). After 3 h, the cells were treated with curcumin (20 µM) or DMSO for 24 h. The β-catenin transcription activity was first normalized to *Renilla* luciferase activity, and expressed as a ratio of pTOP-FLASH/pFOP-FLASH activity. The activity of curcumin treated cells was normalized to activity of vehicle treated cells (considered 100%). Curcumin treatment significantly reduced β-catenin transcription activity in C4-2 cells compared to vehicle treated cells. Mean ± SE, n = 3, *p<0.01. D). Effect of curcumin on transcription of cyclin D1. The transcription of cyclin D1 was analyzed from cells treated with curcumin or vehicle control for 24 h. After reverse transcription of RNA to cDNA, PCR amplification of cyclin D1 or internal control GAPDH was carried out using gene specific primers. The amplified products were resolved on 1% agarose gel. The densitometric quantitation of cyclin D1 band intensities normalized to GAPDH levels is shown in graph. Curcumin treatment reduced the expression of cyclin D1. AU- arbitrary units. E). Immunoblot analyses. Cell lysates prepared from curcumin (20 µM) or DMSO treated C4-2 cells were resolved by SDS-PAGE and processed for immunoblotting using specific antibodies. Curcumin treatment markedly decreased cyclin D1 expression, whereas no effect was observed on the expression of total β-catenin, E-cadherin or Wnt 3a. Representative immunoblots from three experiments are shown.

PKD1 modulates the β-catenin signaling pathway by interacting, phosphorylating and modulating the subcellular localization and inhibiting the transcription activity of nuclear β-catenin [12]. We observed maximal PKD1 activation by curcumin treatment within 1 h (Figure 2B). Transient activation of PKD1 has been shown to have long term downstream cellular effects [12], [17]. Therefore, we further determined the effect of curcumin on membrane β-catenin localization after 24 h of treatment using confocal microscopy (Figure 5A). Higher membrane β-catenin localization along with reduced cytoplasmic β-catenin was observed in cells after 24 h of curcumin treatment (Figure 5A). In addition, 24 h curcumin treatment also altered the subcellular localization of PKD1 (Figure 5A). While in control cells, PKD1 was primarily localized in the cytoplasm, curcumin treated cells exhibited PKD1 localization on the membrane and in the nucleus (Figure 5A).

β-catenin is a critical component of the Wnt signaling cascade. Nuclear β-catenin functions as a co-transcription factor by forming a complex with TCF and enhances the expression of a number of molecules with pro-oncogenic roles, including cyclin D1, c-myc and c-jun. Since PKD1 modulates the subcellular localization of β-catenin and since curcumin mediated PKD1 activation enhanced the membrane localization of β-catenin, we analyzed the effect of this activation on nuclear β-catenin expression using immunoblotting and on β-catenin transcription activity using a luciferase reporter system in C4-2 prostate cancer cells (Figures 5B and 5C). Curcumin treatment significantly reduced the nuclear expression of β-catenin compared to dimethyl sulfoxide (DMSO) treated cells (Figure 5B). The decrease in nuclear β-catenin levels was correlated with the attenuation of β-catenin transcription activity in C4-2 (Figure 5C). In order to determine the downstream effects of the suppressed β-catenin activity, the expression of cyclin D1 was analyzed in curcumin treated C4-2 cells. Interestingly, a marked decrease in the expression of cyclin D1 at mRNA (Figure 5D) and protein levels (Figure 5E) was observed in curcumin treated C4-2 cells. Similar results were also obtained by real-time PCR analysis (data not shown). However, no apparent change was observed in the expression of Wnt 3a, β-catenin and E-cadherin (Figure 5E).

We also determined the effect of curcumin on β-catenin transcription activity in LNCaP cells. Similar to C4-2 cells, curcumin inhibited β-catenin transcription activity (Figure S3A) and markedly decreased the expression of cyclin D1 (Figure S3B) in LNCaP cells.

### Curcumin treatment suppresses oncogenic phenotype in prostate cancer cells

**Figure 6:**
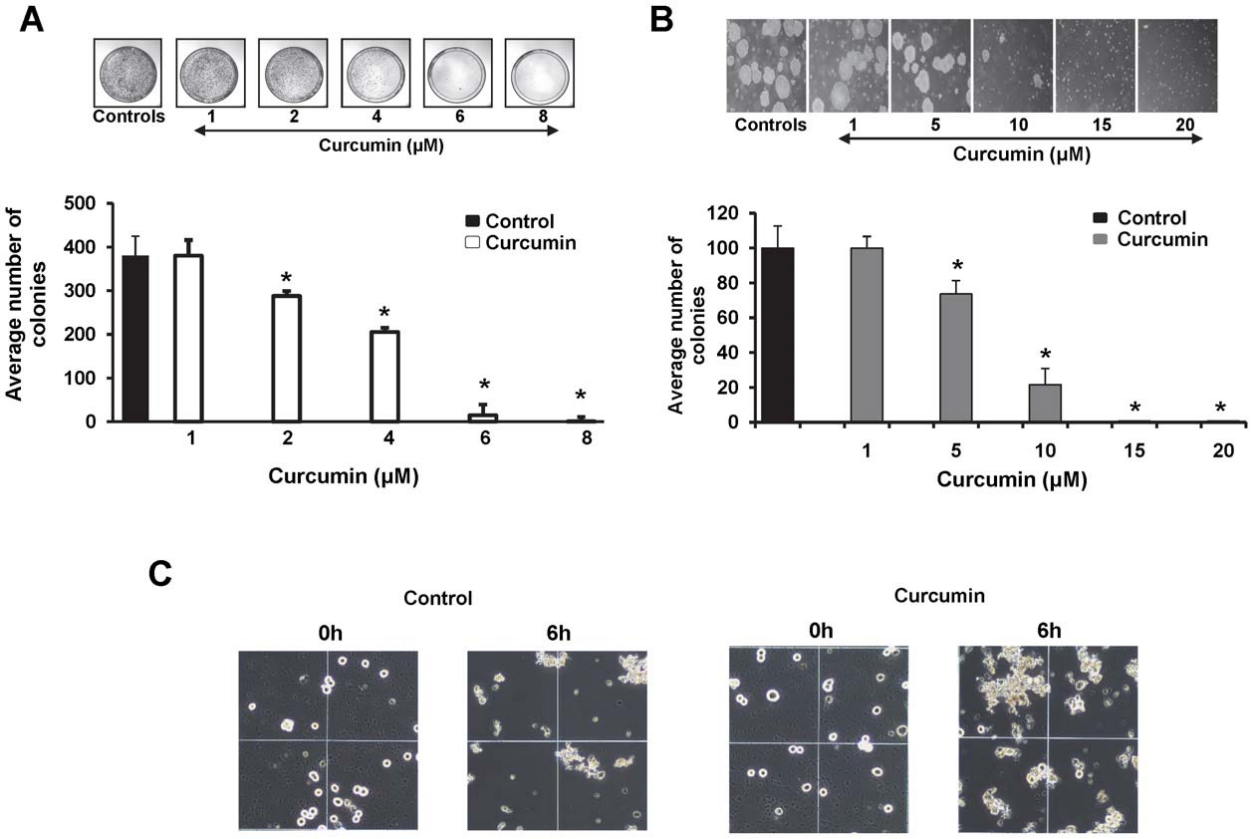
Curcumin treatment attenuates colony formation and cell-cell aggregation. A). Anchorage dependent colony formation assay. C4-2 cells (2000) were plated overnight, treated with indicated concentrations of curcumin for 14 days and examined for their colony forming ability. Representative photographs are shown. Curcumin showed a dose-dependent inhibition in anchorage dependent colony formation assay. Mean ± SE; n = 3; *p<0.05. B). Anchorage independent colony formation assay. C4-2 cells were seeded in 0.3% agarose and treated with varying concentrations of curcumin for 9 days. The number of colonies were counted and plotted. Curcumin treatment inhibited anchorage independent colony formation of C4-2 cells. Mean ± SE; n = 3; *p<0.01. C) Cell-cell aggregation assay. C4-2 cells treated with curcumin (15 µM) or DMSO for 1 h were harvested and assayed for cell-cell aggregation by incubating under gentle shaking conditions at 37°C in the presence of 5 mM CaCl_2_. After 6 h incubation, an aliquot of the reaction mixture was analyzed and photographed for cell-cell aggregation under phase contrast microscope. Larger cell-cell aggregates were observed in curcumin treated cells, compared to DMSO control cells. Original Magnifications 100×.

Enhanced clonogenic potential and decreased cell-cell adhesion are two important characteristics of cancer growth and metastasis. The clonogenic growth characteristic is necessary for the cancer cells to establish a primary tumor and eventually metastasize. Hence, we examined the ability of curcumin to inhibit anchorage dependent and anchorage independent colony formation. Curcumin treatment inhibited anchorage dependent and anchorage independent colony formation in a dose-dependent manner in C4-2 prostate cancer cells (Figure 6A, B). In anchorage independent assays, curcumin treatment not only decreased the number of colonies, but also decreased the size of the colonies (Figure 6B). Similar results were observed in LNCaP cells (Figure S3C, D).

Cell-cell adhesion is facilitated by intercellular cadherin-cadherin interaction. The increased membrane localization of β-catenin strengthens the cadherin-catenin interactions and thus enhances cell-cell interactions [12]. In order to determine the effect of enhanced membrane localization of β-catenin, curcumin treated C4-2 and PKD1 overexpressing C4-2 cells (C4-2-PKD1) were assessed for cell-cell aggregation. Curcumin treated C4-2 cells formed larger cell-cell aggregates compared to the control treated cells (Figure 6C). Interestingly, while C4-2-PKD1 formed large cell aggregates, much larger cell-cell aggregates were observed upon curcumin treatment of these cells (Figure S4). These data implicate a role of curcumin mediated activation of PKD1 in cell-cell aggregation.

### Curcumin inhibits cell motility through PKD1 mediated cofilin phosphorylation

**Figure 7:**
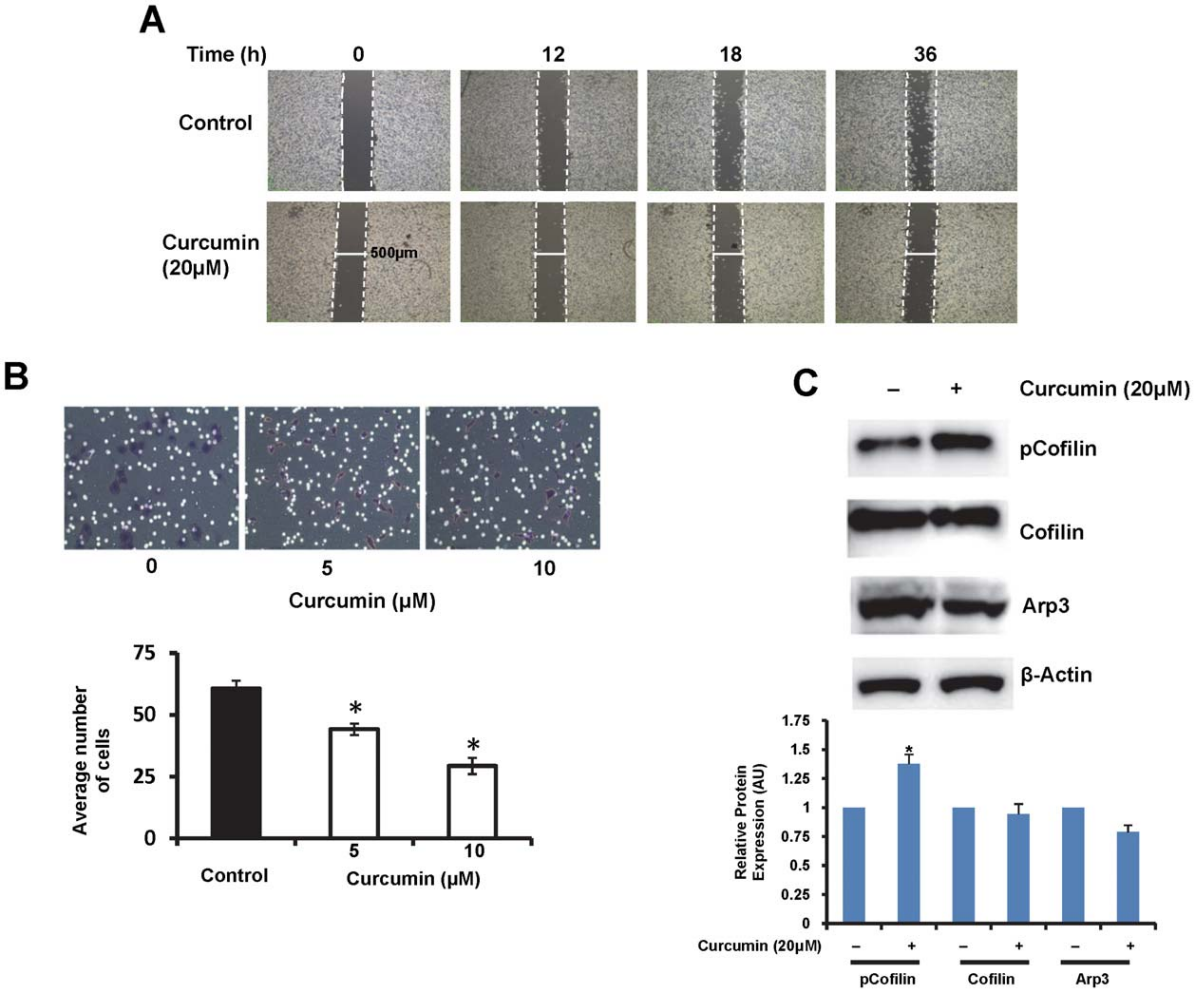
Curcumin treatment inhibits cell motility through phosphorylation of cofilin. A). Scratch assay. C4-2 cells were grown, until confluent, in plates containing IBIDI inserts. The inserts were removed from the plates to generate gaps (solid white lines show width of the gap; dashed lines border the gap) and phase contrast images of the same area of the gaps were taken at varying time intervals in the presence or absence of 20 µM curcumin. Curcumin treatment inhibited motility of C4-2 prostate cancer cells. B). Boyden's chamber assay. Equal numbers of C4-2 cells were seeded on the Boyden's chambers and incubated in the presence DMSO or curcumin (20 µM) for 24 h. Migrated cells were fixed, stained, counted and graphed. Curcumin inhibited motility of C4-2 cells. Mean ± SE; n = 3; *p<0.05. C). Effect of curcumin on the expression of actin remodeling proteins. Total cell lysates prepared from curcumin (20 µM) or DMSO treated C4-2 cells were processed for immunoblotting using specific antibodies. The densitometric quantitation of protein bands normalized to β-actin level is shown in graph. Curcumin treatment induced a marked increase in the expression of inactive phospho-cofilin compared to DMSO treated control cells. Minor change was also observed in the expression of Arp3. AU- arbitrary units.

Cancer cells usually exhibit enhanced cellular motility to facilitate metastasis. Therefore, the ability of a chemopreventive or chemotherapeutic agent to inhibit cell motility will aid in preventing cancer metastasis. Hence we investigated the effect of curcumin on cell motility using a ‘wound healing’ and Boyden's chamber assays. Compared to control, curcumin treatment inhibited cellular motility of C4-2 prostate cancer cells in both assays (Figure 7A, B). A similar effect on cellular motility was also observed in LNCaP cells (Figure S3E).

The highly coordinated process of actin remodeling underlies the process of cellular motility. This remodeling at the growing front requires the orchestrated action of a number of molecules involved in the F-actin reorganization. The actin related proteins (Arp) play an important role in the branching of the actin filament. The protein cofilin is an actin monomer generating molecule that is involved in actin remodeling. Cofilin is activated by slingshot (SSH) phosphatase mediated dephosphorylation reaction and is inactivated by LIM Kinase (LIMK) mediated phosphorylation reactions [2], [21]. PKD1 is intricately involved in inhibiting cell motility by interacting, phosphorylating and inhibiting the functions of many motility related proteins including a slingshot 1 like (SSH1L) phosphatase [21]. Since curcumin activated PKD1, we sought to investigate the effect of curcumin on the activity and levels of cofilin and Arp3 protein by immunoblotting. Curcumin treatment increased the levels of inactive phospho-cofilin in C4-2 prostate cancer cells with little or no effect on the expression of the total protein (Figure 7C). Curcumin treatment also caused a slight decrease in the expression of Arp3 protein. These data suggest a potential role of PKD1 in curcumin mediated inhibition of cell motility *via* cofilin phosphorylation.

### *In vivo* effects of curcumin on prostate cancer growth

**Figure 8:**
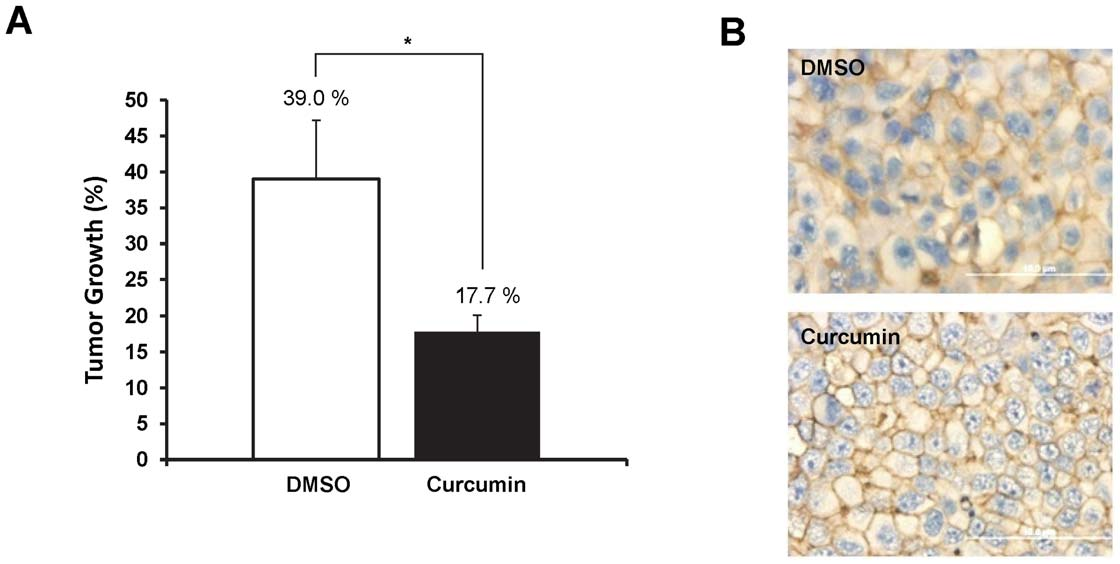
Curcumin inhibits prostate cancer growth in xenograft mouse model. A) Effect of curcumin on prostate cancer growth. C4-2 prostate cancer cells were used to generate xenografts in male nude mice. Following tumor development, the mice were treated intra-tumorally with curcumin (n = 4) or DMSO (n = 3). The rate of tumor growth was measured after 7 day and the percent tumor growth following treatment was graphed. Curcumin effectively inhibits prostate cancer growth. B) Effect of curcumin on β-catenin localization. Tumor tissues from curcumin or control treated mice were processed for IHC staining using anti-β-catenin antibody. Enhanced staining of membranous β-catenin was observed in curcumin treated mice compared to control mice. Original Magnifications 400×.

A xenograft mouse model was used to examine the *in vivo* effect of curcumin on prostate tumor growth and β-catenin subcellular localization. Nude mice were subcutaneously inoculated with androgen-independent C4-2 cells. Following tumor development, the mice were administered intra-tumoral injections of curcumin or vehicle control. On day 7, the tumor volumes were measured and the rate of tumor growth following curcumin treatment was determined. Curcumin efficiently inhibited tumor growth by over two folds compared with the control-treated mice (*p<0.05) (Figure 8A). In addition, we observed change in β-catenin subcellular localization in curcumin treated tumor tissues (Figure 8B), similar to *in vitro* observations (Figure 3 and 5A). These results suggest that curcumin inhibits prostate tumor growth by modulating β-catenin functions.

## Discussion

The dysregulation of PKD1, a serine-threonine kinase, has been associated with cancer progression [2], [4], [6]. PKD1 is expressed at the highest level in the prostate gland and plays a critical role in the normal physiology of the prostate [9], [10]. Previous work from our laboratory has revealed the association of PKD1 downregulation with the progression of prostate cancer [3], [11]. Our previous work has also illuminated the role of PKD1 in E-cadherin phosphorylation, modulation of cell motility and cell-cell aggregation in prostate cancer cells [13]. In addition, we have shown PKD1 to interact with, phosphorylate and modulate the function of β-catenin [12]. The natural macrolactone Bryostatin 1 activates PKD1 in prostate cancer cells [12]. The activated PKD1 phosphorylates and translocates nuclear β-catenin from the nucleus resulting in the inhibition of β-catenin/TCF transcriptional activity [12]. Thus natural compounds that modulate PKD1 activation might help in prevention and treatment of prostate cancer. Curcumin is a natural compound that is currently in clinical trials for prevention and treatment of various cancers [33]–[35]. In this study, we have demonstrated that curcumin activates PKD1, attenuates β-catenin/TCF transcriptional activity and enriches membrane β-catenin resulting in the suppression of prostate cancer growth.

β-catenin is a multifunctional protein that plays an important role in ontogenesis and oncogenesis. In combination with TCF and p300, it functions as a transcription factor in the Wnt signaling pathway [36]. In addition, β-catenin, along with E-cadherin functions at the cell membrane as a critical component of the adherens junction to enhance cell-cell adhesion [37]. Thus the dysregulation of β-catenin has been associated with the development of many types of cancers, including prostate cancer [36], [38], [39]. We have previously shown a novel mechanism of β-catenin regulation through the action of PKD1 [12]. Herein, we have revealed that curcumin activates PKD1 within 1 h of treatment (Figure 2B). Additionally, using a reporter luciferase assay, we have demonstrated that the β-catenin activity is inhibited by curcumin, following PKD1 activation (Figure 5C).

Since β-catenin is an important signaling molecule, its functions are regulated by multiple pathways [40]. While curcumin has been shown to inhibit β-catenin/TCF transcription activity [41], its precise molecular mechanisms are not fully known. Herein, we for the first time demonstrated that curcumin attenuates β-catenin/TCF transcription activity *via* activation of PKD1 in prostate cancer cells. Studies have also reported inhibition of β-catenin transcriptional activity upon curcumin treatment in colon cancer cells via caspase-3 mediated degradation of β-catenin [42]. However, we did not observe a marked decrease in overall β-catenin expression levels in prostate cancer cell lines. This suggests a possible cell type variation in curcumin mediated inhibition of β-catenin transcription activity. Interestingly, curcumin treatment alters the subcellular localization of PKD1 in prostate cancer cells (Figure 3C). Compared to control cells that revealed predominant PKD1 localization in the cytoplasm, curcumin treated cells showed PKD1 localization predominantly on the cell membrane and in the nucleus, with very low expression in the cytoplasm (Figure 5A). The enhanced presence of PKD1 in the nucleus following curcumin treatment suggests the role of PKD1 in the attenuation of nuclear β-catenin/TCF activity, probably by phosphorylation and/or shuttling of nuclear β-catenin out to the nucleus. We also showed that a decrease in nuclear β-catenin transcription activity results in the lowered expression of cyclinD1, a downstream oncogene of β-catenin/TCF transcription activity. Our *in vitro* (Figure 6) and *in vivo* (Figure 8) studies showed that curcumin effectively attenuated prostate cancer growth. Thus, since active PKD1 is involved in shuttling of nuclear β-catenin out of the nucleus, our study suggests a novel mechanistic role for curcumin mediated attenuation of β-catenin/TCF activity and prostate cancer growth through activation of PKD1. The activation of PKD1 and the reduction of β-catenin transcriptional activity by curcumin may also impact androgen receptor (AR) signaling in prostate cancer, since both PKD1 and β-catenin modulate AR function. Although previous studies have demonstrated that curcumin treatment modulates the levels and transcription activity of AR [43]–[45], the activation of PKD1 by curcumin and the inhibition of β-catenin activity might be another mechanism for the regulation of AR function and prostate cancer growth.

The cadherin-catenin complex forms the adhesion junction that is essential for maintaining cell-cell adhesion. The transmembrane E-cadherins is linked to the actin cytoskeleton through its interaction with β-catenin, α-catenin and γ-catenin [37]. The loss of cell-cell adhesion is a critical factor responsible for cancer metastasis. Previous work from our laboratory and others has revealed the regulation of both E-cadherin and β-catenin by PKD1 [13], [18], [39]. An increased level of β-catenin or E-cadherin on the membrane facilitates enhanced cell-cell aggregation. We have previously shown a novel mechanism of β-catenin regulation through PKD1 [12]. In this study we showed that curcumin treatment enriched the levels of membrane β-catenin (Figure 3 and Figure S2) and this change in the subcellular localization of β-catenin is mediated by PKD1 upon curcumin treatment (Figure 4). Additionally, we showed that curcumin treatment enhanced cell-cell aggregation (Figure 6C and Figure S4), probably by enhanced β-catenin localization on the cell membrane. The specificity of the role of PKD1 in these processes was confirmed by PKD1 siRNA and exogenous overexpression of PKD1 in C4-2 cells. This change in β-catenin subcellular localization was also reflected in xenograft mouse studies (Figure 8B), implicating the role of curcumin mediated activation of PKD1 in prostate cancer.

Additionally, in this study we have demonstrated a novel molecular mechanism responsible for the inhibition of cell motility by curcumin treatment. Our results revealed that curcumin inhibits cell motility by decreasing the levels of active cofilin. Cofilin is a protein necessary for actin remodeling, which is a molecular process essential for cell motility. This dynamic and complex process of actin remodeling involves the coordinated action of a number of proteins to actively balance the polymerization and growth of fibrous actin with depolymerization/severing of the actin polymers [2], [21]. Cofilin activity is tightly regulated by phosphorylation and dephosphorylation mechanisms. Cofilin is inactivated by the phosphorylating action of LIMK, while the SSH enzyme converts inactive cofilin to active form through its dephosphorylating activity [21]. The cell further finely controls this process by regulating the activities of LIMK and SSH through phosphorylation and dephosphorylation reactions. PKD1 has been shown to play a critical role in inhibiting cellular motility through inhibition of SSH activity and activation of LIMK activity [20], [22], [23]. Herein, we showed that curcumin treatment, which activates PKD1, results in the accumulation of inactive phospho-cofilin to inhibit cell motility.

**Figure 9:**
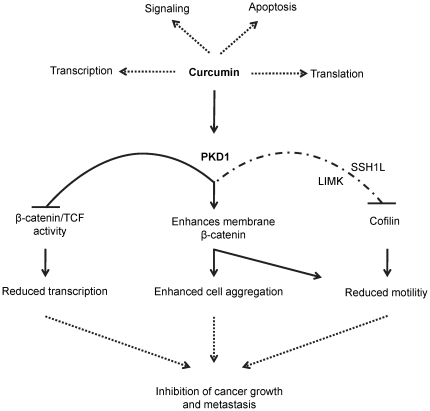
Schematic diagram showing possible signaling mechanisms modulated by curcumin mediated PKD1 activation. Curcumin modulates a number of molecular pathways within the cancer cells including PKD1 signaling. Curcumin may suppress prostate cancer growth and metastasis by activating PKD1, which in turn may inhibit cell growth through the inhibition of β-catenin/TCF transcription activity, enhance cell-cell aggregation *via* enhanced translocation of β-catenin to the cell membrane and inhibit cell motility either directly by enhancing cell-cell aggregation and/or phosphorylating and inhibiting the function of sling shot 1 like (SSH1L) phosphatase or indirectly (dashed lines) by negatively regulating the expression of active cofilin *via* indirectly activating LIM kinase (LIMK).

In conclusion, our study elucidates a new molecular paradigm involving PKD1 signaling in mediating the anti-cancer effects of curcumin (Figure 9). The nutraceutical compound curcumin may act as a chemo-preventive agent to inhibit or delay the onset of prostate cancer *via* the activation of PKD1. Additionally, curcumin treatment could also synergize conventional chemotherapy by activating PKD1 and inhibiting β-catenin transcriptional activity. Further, activation of PKD1 by curcumin may prevent metastasis by enhancing cell-cell adhesion and inhibiting cell motility. This study suggests a novel molecular mechanism of curcumin mediated prostate cancer prevention/treatment.

## Materials and Methods

### Materials

Media containing glutamine (RPMI 1640, High glucose DMEM), fetal bovine serum (FBS), 100 mM sodium pyruvate solution and 1× antibiotic and antimycotic solutions were purchased from Hyclone (Hyclone Laboratories, South Logan, UT). OPTI-MEM reduced serum growth media was purchased from Invitrogen (Life Technologies, Carlsbad, CA). All other chemicals were purchased from Sigma (Sigma-Aldrich, St. Louis, MO) unless mentioned otherwise.

### Cell culture

All the cells were aseptically handled and grown at 37°C in a humidified incubator containing 5% CO_2_. C4-2 (UroCor, Oklahoma City, OK), PC3, and LNCaP prostate cancer cells (ATCC, Manassas, Virginia) and C4-2 cells overexpressing PKD1 [13] were maintained in RPMI 1640 media containing 1 mM sodium pyruvate, 2.5 mM glutamine, 10% heat-inactivate FBS (Hyclone) and 1× antibiotic and antimycotic solution. Early passage LNCaP cells (passage 32–37) were used for experiments. DU145 prostate cancer cells (ATCC) was maintained in DMEM media containing 1 mM sodium pyruvate, 2.5 mM glutamine, 10% heat-inactivate FBS (Hyclone) and 1× antibiotic and antimycotic solution.

### Antibodies

The mouse monoclonal β-catenin antibody is a generous gift of Dr. Keith Johnson (University of Nebraska Medical Center, Omaha, Nebraska) and has been described previously [46]. Rabbit polyclonal PKD1 antibody (C-20), Histone H1 (Santa Cruz Biotechnologies, Santa Cruz, CA), rabbit monoclonal PKD1, rabbit monoclonal phospho-PKD1 (744.748), rabbit monoclonal phospho-PKD1 (916), E-cadherin, cofilin, phospho-cofilin, Arp3, Wnt3a, c-Myc (Cell Signaling Technologies, Danvers, MA), β-actin (Sigma-Aldrich and cyclinD1 (Santa Cruz Biotechnologies) were commercially procured.

### Cell proliferation assay

The effect of curcumin on cell proliferation was determined by MTS assay using CellTiter96 Aqueous One Solution reagent (Promega, Madison, WI). Briefly, prostate cancer cells (5000 cells/well) were plated in 100 µl RPMI in 96 well tissue culture plates and incubated for 12 h. Curcumin was dissolved in DMSO and diluted in tissue culture media. The cells were treated with varying concentrations of curcumin or equivalent amounts of vehicle control (DMSO) and incubated for 48 h. The cell proliferation was determined by adding 20 µl of MTS reagent, incubating for 2 h at 37°C and measuring the absorption at 490 nm using a SPECTRA max Plus plate reader (Molecular Devices, Sunnyvale, CA). The percent proliferation in curcumin treated cells was determined by normalizing to cells treated with equivalent amount of vehicle considered (100%). The results expressed are average of three independent experiments.

### Immunoblotting

Prostate cancer cells were grown and treated with curcumin (20 µM) or DMSO for immunoblot analysis as described earlier [12]. Briefly, cells were washed, and lysed in 2× SDS lysis buffer. Following normalization of protein concentrations of the cell supernants using SYPRO Orange dye (Life Technologies), the protein samples were electrophoretically resolved on a 4–20% SDS-PAGE and transferred onto a PVDF membrane (Bio-Rad Laboratories, Hercules, CA). The blots were probed for various proteins using specific primary antibodies. Following incubation with HRP-labelled secondary antibody (Promega), protein bands were detected with Lumi-Light Plus chemi-luminescent reagent (Roche, Indianapolis, IN).

### Immunofluorescence

Immunofluorescence was performed as described earlier [12]. Briefly, C4-2 and LNCaP prostate cancer cells (passage 34–37) (1×10^5^/well) were grown on glass coverslips and treated with curcumin (20 µM) or DMSO for varying time points. The cells were fixed in 2% paraformaldehyde, permeabilized, blocked and then incubated with primary antibody. Following washing, the cells were incubated with Alexa488 or Cy3 labeled secondary antibody. After washing, the cover slips were mounted on glass slide using aqueous antifade medium (Vector Laboratories, Burlingame, CA). The slides were analyzed by confocal laser scanning microscopy (Olympus FV1000 Laser Scanning Microscope, Olympus, Japan).

### Silencing of PKD1 by siRNA

The expression of PKD1 in prostate cancer cells was blocked using synthetic siRNA as mentioned earlier [12]. Briefly, C4-2 cells were transfected with 25 nM synthetic siRNA duplexes (sense 5-GGAAGGAAAUAUCUCAUGAUU, antisense 5-PUCAUGAGAUAUUUCCUUCCUU; Life Technologies) for 24 h to silence PKD1 expression using Dharmafect Transfection Reagent (Life Technologies). The transfected cells were then used for further experiments. For negative control experiments, cells transfected with 25 nM scrambled control sense siRNA (5-UAGCGACUAAACACAUAA; Life Technologies) were used.

### Reporter assay for detection of β-catenin transcription activity

A luciferase reporter assay system was used to measure β-catenin/TCF transcription activity as previously mentioned with slight modifications [12]. Briefly, actively growing cancer cells were plated overnight in 12 well plates (2×10^5^ cells/well) in normal growth medium. After replacing the growth medium with reduced serum OPTI-MEM media for one hour, the cells were transiently transfected with TCF-firefly luciferase reporter construct containing either wildtype TCF promoter binding sites (pTOP-FLASH) or mutant TCF promoter binding sites (pFOP-FLASH) (generous gift from Dr. R. Moon, Washington University) and co-transfected with *Renilla* luciferase construct (pRL-TK) (Promega) to normalize for transfection efficiency. Three hours post-transfection, the cells were treated with either 20 µM curcumin or equivalent amount of control vehicle (DMSO) for 24 h and cell lysates were prepared using luciferase lysis buffer (25 mM Tris phosphate pH 7.8, 0.1% Triton X-100, 1 mM DTT). The firefly luciferase activity and *Renilla* luciferase activity were assayed in a two step process using the Dual Glo reagents (Promega) according to the manufacturer's instructions and the signals measured in GLOMAX 96 microplate luminometer (Promega). The β-catenin/TCF transcription activity was determined by first normalizing the firefly luciferase acitivty to that of *Renilla* luciferase acitivity and finally calculating the ratio of TOP-FLASH signal to FOP-FLASH signal. The β-catenin/TCF transcription activity of curcumin treated cells was expressed in percentage after normalizing to activity in vehicle treated cells (considered 100%). Non-paired t-test was used to determine the value of statistical significance (*p value).

### RNA isolation and reverse transcription PCR

Total RNA was isolated from prostate cancer cells using an RNA isolation kit (Qiagen Inc, Valencia, CA). The RNA (2 µg) was reverse transcribed (RT) to cDNA using Applied Biosystems High capacity RNA to c-DNA kit according to manufacturer's instruction (Life Technologies). The RT reaction was carried out in a thermocycler for 1 h at 30°C, followed by inactivation of the enzyme at 95°C for 5 min. This cDNA was used for amplification of specific genes in a final volume of 25 µl using 200 nM gene specific primers (cyclin D1: Forward primer- 5′-CCG CTG GCC ATG AAC TAC CT; Reverse primer- 5′-ACG AAG GTC TGC GCG TGT T), 200 µM dNTP, 1.5 mM MgCl_2_, and 1.25 unit Taq DNA polymerase (Eppendorf Master Mix, Eppendorf, Hamburg, Germany). The PCR cycling conditions used included a denaturation step at 95°C for 4 min; 25 cycles of denaturation at 95°C for 30 sec, annealing at 53°C for 30 sec and extension at 72°C for 1 min; and a final extension at 72°C for 10 min. The amplification of GAPDH (Forward primer- 5′-GAA GGT GAA GGT CGG AGT C; Reverse primer- 5′-GAG GGA TCT CGC TCC TGG AAG A) was used as internal control. The amplified samples were resolved on 1% agarose gel and imaged.

For real time PCR analysis, the cDNA generated from curcumin treated samples were amplified with SYBR Green PCR master mix (Applied Biosystems) using a 7500 Real-Time PCR System (Applied Biosystems). Each reaction was performed in duplicate and the amplification of β2-microglobulin (Forward primer- 5′-AGA TGA GTA TGC CTG CCG TGT GAA; Reverse primer- 5′-TGC TGC CAT GTC TCG ATC CCA) was used as internal control. The specificity of the PCR reaction was confirmed by examining the melting curve for a single peak. The results were analyzed using the 7500 System SDS software (Applied Biosystems). The results were first normalized to control for the β2-microglobulin gene and percent change in the expression levels of the drug treated sample was compared to vehicle treated samples. Non-paired t-test was used to determine the value of statistical significance (*p value).

### Anchorage dependent colony formation assay

This was performed as described earlier [47] with slight modifications. Cells (2×10^3^) were plated in 100 mm cell culture dishes. Following overnight incubation, the cells were treated with varying concentrations of curcumin in fresh media. After 12–14 days, the plates were washed, fixed and stained with 0.05% crystal violet solution. Visible colonies were counted and plotted against curcumin concentrations. Non-paired t-test was used to determine the statistical significance value (*p value).

### Anchorage independent colony formation assay

Anchorage independent assay was performed as described earlier [45] with slight modifications. In this two layered agarose assay, the bottom agarose layer was cast by adding 1 ml of 0.6% agarose medium/well in 6 well plates. The cells (4×10^4^) were seeded in 0.35% agarose, treated with varying concentrations of curcumin or DMSO and cultured for 10 days. The plates were stained with 0.05% crystal violet and formed colonies (cluster of >30cells) were imaged using a phase contrast microscope. The numbers of colonies were counted and average number of colonies was plotted against curcumin concentrations.

### Aggregation assay

The aggregation assay was performed as described earlier [48]. In brief, tissue culture plates containing actively growing cells were treated with varying concentrations of curcumin or appropriate amount of vehicle control for 1 h. The cells were trypsinized with 0.01% trypsin-EDTA, washed with PBS containing 5 mM CaCl_2_, and then resuspended (1×10^6^ cells/ml) in RPMI media containing 5 mM CaCl_2_ in polystyrene tubes. The tubes were incubated in a shaker for 6 h at 37°C at low speed (20 rpm) and the cellular-aggregations in suspensions were observed under a phase contrast microscope.

### Cell motility assay

The motility assay was performed using a scratch assay as previously described [49]. Briefly, cells (0.5×10^6^cells/plate) were cultured in 35 mm plates containing IBIDI cell culture inserts (Integrated Bio Diagnostics, München, Germany), until confluent. The media was carefully removed and replaced with 2 ml media containing either 20 µM curcumin or DMSO for 1 h. The inserts were carefully removed under asceptic conditions (creating a 500 mm cell free area) and phase contrast images of three cell free areas were taken using EVOS microscope (Advanced Microscope Group, Bothell, WA) at varying time intervals.

Boyden's chamber motility assay: The Boyden's chamber assay was carried out as described elsewhere [39]. Briefly, cells, pretreated for 6 h with 5 or 10 µM curcumin or DMSO were harvested and suspended at 0.3×10^6^ cells/1 ml in RPMI media containing 1% FBS and curcumin or DMSO. These cells were seeded on the Boyden's chambers and placed in 6 well plates containing identical medium with higher concentration of FBS (10%). The chemo-tactic gradient generated by the different FBS concentration between the upper and lower chamber induces the cells to migrate. Following 24 h incubation, the non-migrated cells were cleaned from the upper chamber and the migrated cells, on the lower side of the membrane were fixed (100% ethanol for 30 min), stained (0.05% crystal violet for 30 min) and the phase contrast images were using EVOS microscope. The migrated cells were counted and the average number of migrated cells was graphed.

### Tumor xenograft study

Six-week-old male athymic nude (nu/nu) mice (Charles River Laboratories, Wilmington, MA) maintained in pathogen-free conditions were used for this study as described earlier [50]. Briefly, C4-2 cells (10×10^6^ cells per mouse in 200 µL) mixed with 100 µl Matrigel (BD Biosciences, Sparks, MD) were injected sub-cutaneously (s.c) into the flank of the left hind limb. The animals were periodically monitored for tumor development. On day 40, the mice were randomly distributed into two groups and intra-tumorally injected with curcumin (25 µg/mouse) or vehicle control (DMSO). The tumor volume was measured after 7 days post intra-tumoral injection. The tumor growth was measured using a digital Vernier caliper and the tumor volume was calculated using the ellipsoid volume formula, tumor volume (mm^3^) = *π*/6×*L*×*W*×*H*, wherein *L* is length, *W* is width, and *H* is height. Mice were sacrificed and the tumors were fixed in formalin. These procedures were carried out following approval by the Sanford Research/University of South Dakota Institutional Animal Care and Use Committee.

### Immunohistochemical (IHC) analysis of prostate xenograft tumors

IHC analysis of formalin fixed, paraffin embedded xenograft mouse tumors (5 µM sections) was performed as previously described [50]. Briefly, the tumor tissues were deparaffinized, rehydrated, treated with 0.3% hydrogen peroxide and processed for antigen retrieval using heat-induced technique. Following blocking with background sniper (Biocare Medical, Concord, CA), the samples were processed for staining with anti-β-catenin antibody. β-catenin expression was detected using MACH 4 Universal HRP Polymer detection kit (Biocare Medical) and 3,3′-diaminobenzidine (DAB substrate kit, Vector Laboratories). The slides were counterstained with hematoxylin, dehydrated, mounted with Vectamount (Vector Laboratories) and visualized using an Olympus BX 41 Microscope (Olympus Corporation, Japan).

## Supporting Information

Figure S1 **Activation of PKD1 by Curcumin.** A). Effect of curcumin on phospho PKD1 levels. C4-2 cells were treated with 20 µM curcumin for varying time points. The cell lysates were resolved by SDS-PAGE and processed for immunoblotting using phospho PKD1 antibody. The densitometric analysis of phospho PKD1 normalized to β-actin levels is shown in graph. Curcumin activates PKD1 by 1 h and remains active until 3 h. At 24 h, however, a slight decline in phosphorylation status was observed. B). Curcumin activates exogenously expressed PKD1. C4-2 cells overexpressing PKD1 (C4-2-PKD1 cells) were treated with 20 µM curcumin, for varying time points and the cell lysates were processed for immunoblotting using phospho PKD1 antibody. Quantitation of the pPKD1 levels normalized to β-actin is shown in graph. Curcumin treatment induced maximal PKD1 activation/phosphorylation by 1 h. C). Curcumin activates PKD1 in LNCaP cells. Cell lysates of LNCaP cells treated with 20 µM curcumin for varying time points were processed for immunoblotting using phospho PKD1 antibody. Quantitation of protein band is shown in graph. Curcumin treatment induced maximal PKD1 activation/phosphorylation by 50–60 min. AU- arbitrary units.
(TIF)

Figure S2 **Curcumin treatment enhances membrane** β**-catenin in LNCaP prostate cancer cells.** A). LNCaP cells were cultured on coverslips overnight in 12 well plates and treated with DMSO or curcumin (20 µM) for 1 h. The cells were processed for immunostaining using anti-β-catenin (red) and PKD1 (green) antibodies and counter-stained with DAPI (blue). Enhanced membranous β–catenin staining was observed on cell surface at 1 h of curcumin treatment, compared to control cells treated with vehicle (DMSO).
(TIF)

Figure S3 **Effect of curcumin treatment on LNCaP prostate cancer cells.** A). Effect of curcumin on β-catenin transcription activity in LNCaP prostate cancer cells. A luciferase based reporter assay system was used to measure the β-catenin transcription activity in LNCaP cells, as described in materials and methods and Figure 5. The β-catenin activity of curcumin treated cells was normalized to the activity of vehicle treated cells (considered 100%). Curcumin treatment significantly reduced β-catenin transcription activity in LNCaP cells compared to vehicle treated cells. Mean ± SE, n = 3, *p<0.01. B). Effect of curcumin on cyclin D1 expression in LNCaP cells. RNA was isolated from LNCaP cells treated with curcumin or vehicle control for 24 h and processed for RT-PCR using cyclin D1 and GAPDH specific primers. The densitometric quantitation of cyclin D1 normalized to GAPDH levels is shown in graph. Curcumin treatment specifically reduced the levels of cyclin D1 gene compared to internal control. AU- arbitrary units. C). Anchorage dependent colony formation assay. LNCaP cells (2000) were plated overnight, treated with indicated concentrations of curcumin for 14 days and examined for their colony forming ability. Curcumin showed a dose-dependent inhibition in anchorage dependent colony formation assay. Mean ± SE; n = 3; *p<0.05. D). Anchorage independent colony formation assay. LNCaP cells were seeded in 0.3% agarose and treated with varying concentrations of curcumin for 9 days. The number of colonies were counted and plotted. Curcumin treatment inhibited anchorage independent colony formation of C4-2 cells. Mean ± SE; n = 3; *p<0.01. E) Boyden's chamber assay. Equal numbers of LNCaP cells were seeded on the Boyden's chambers and incubated in the presence DMSO or curcumin for 24 h. Migrated cells were fixed, stained, counted and graphed. Curcumin inhibited motility of LNCaP cells. Mean ± SE; n = 3; *p<0.05.
(TIF)

Figure S4 **Effect of curcumin treatment on cell-cell aggregation in C4-2-PKD1 cells.** C4-2 cells overexpressing PKD1 were treated with curcumin (15 µM) or DMSO for 1 h, harvested and assayed for cell-cell aggregation by incubating under gentle shaking conditions at 37°C in the presence of 5 mM CaCl_2_. After 6 h incubation, an aliquot of the reaction mixture was photographed for cell-cell aggregation under phase contrast microscope. C4-2-PKD1 cells formed larger cell-cell aggregates than control treatment. Original magnifications 100×.
(TIF)
