# Supplementary figures and images for: Curcumin Attenuates β-catenin Signaling in Prostate Cancer Cells through Activation of Protein Kinase D1
Source: PLoS One. 2012 Apr 16;7(4):e35368. doi: 10.1371/journal.pone.0035368 (PMC3327669; doi:10.1371/journal.pone.0035368)

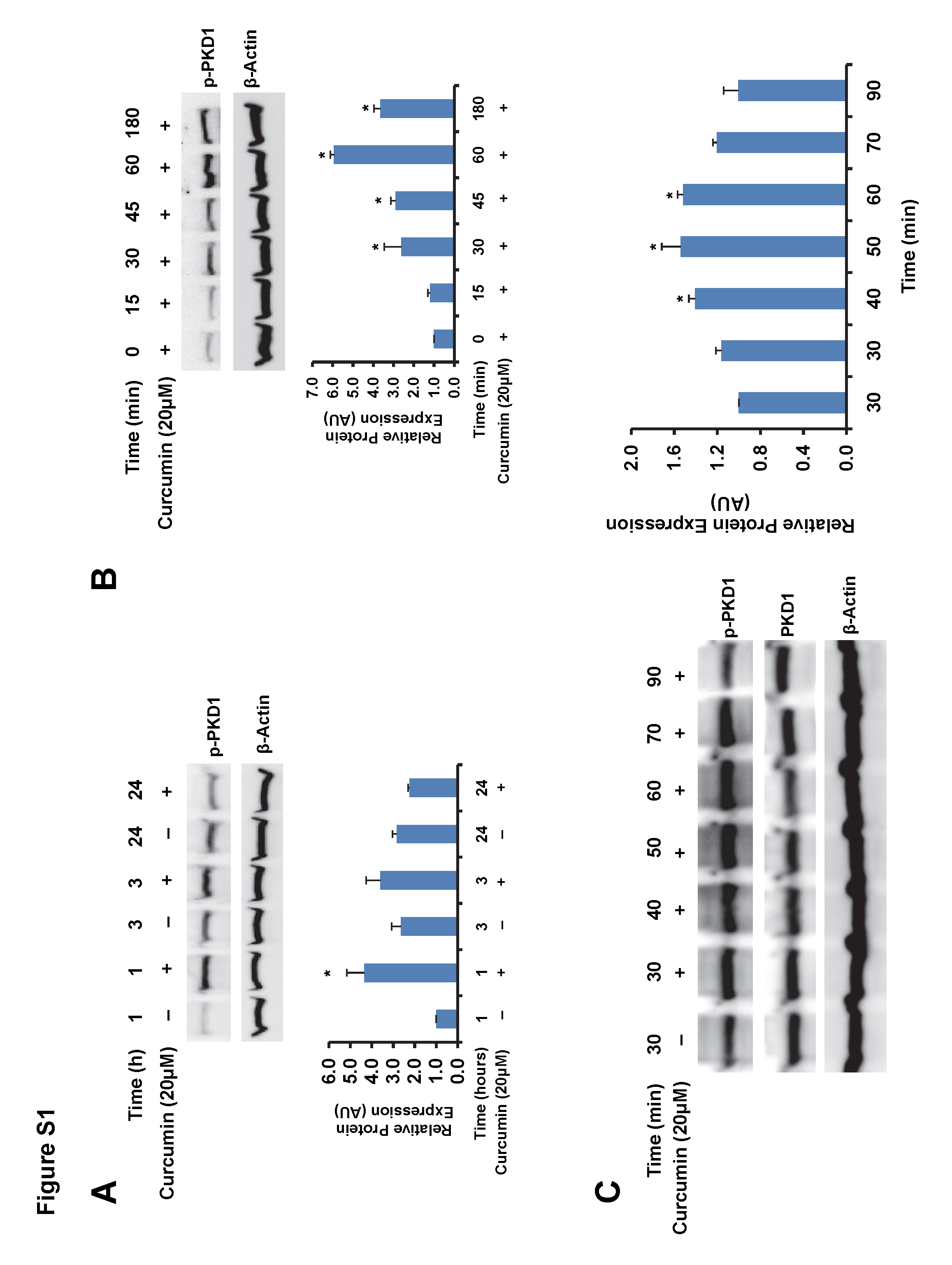

Supplement: Figure S1 — Activation of PKD1 by Curcumin. A). Effect of curcumin on phospho PKD1 levels. C4-2 cells were treated with 20 µM curcumin for varying time points. The cell lysates were resolved by SDS-PAGE and processed for immunoblotting using phospho PKD1 antibody. The densitometric analysis of phospho PKD1 normalized to β-actin levels is shown in graph. Curcumin activates PKD1 by 1 h and remains active until 3 h. At 24 h, however, a slight decline in phosphorylation status was observed. B). Curcumin activates exogenously expressed PKD1. C4-2 cells overexpressing PKD1 (C4-2-PKD1 cells) were treated with 20 µM curcumin, for varying time points and the cell lysates were processed for immunoblotting using phospho PKD1 antibody. Quantitation of the pPKD1 levels normalized to β-actin is shown in graph. Curcumin treatment induced maximal PKD1 activation/phosphorylation by 1 h. C). Curcumin activates PKD1 in LNCaP cells. Cell lysates of LNCaP cells treated with 20 µM curcumin for varying time points were processed for immunoblotting using phospho PKD1 antibody. Quantitation of protein band is shown in graph. Curcumin treatment induced maximal PKD1 activation/phosphorylation by 50–60 min. AU- arbitrary units. (TIF) [file pone.0035368.s001.tif]

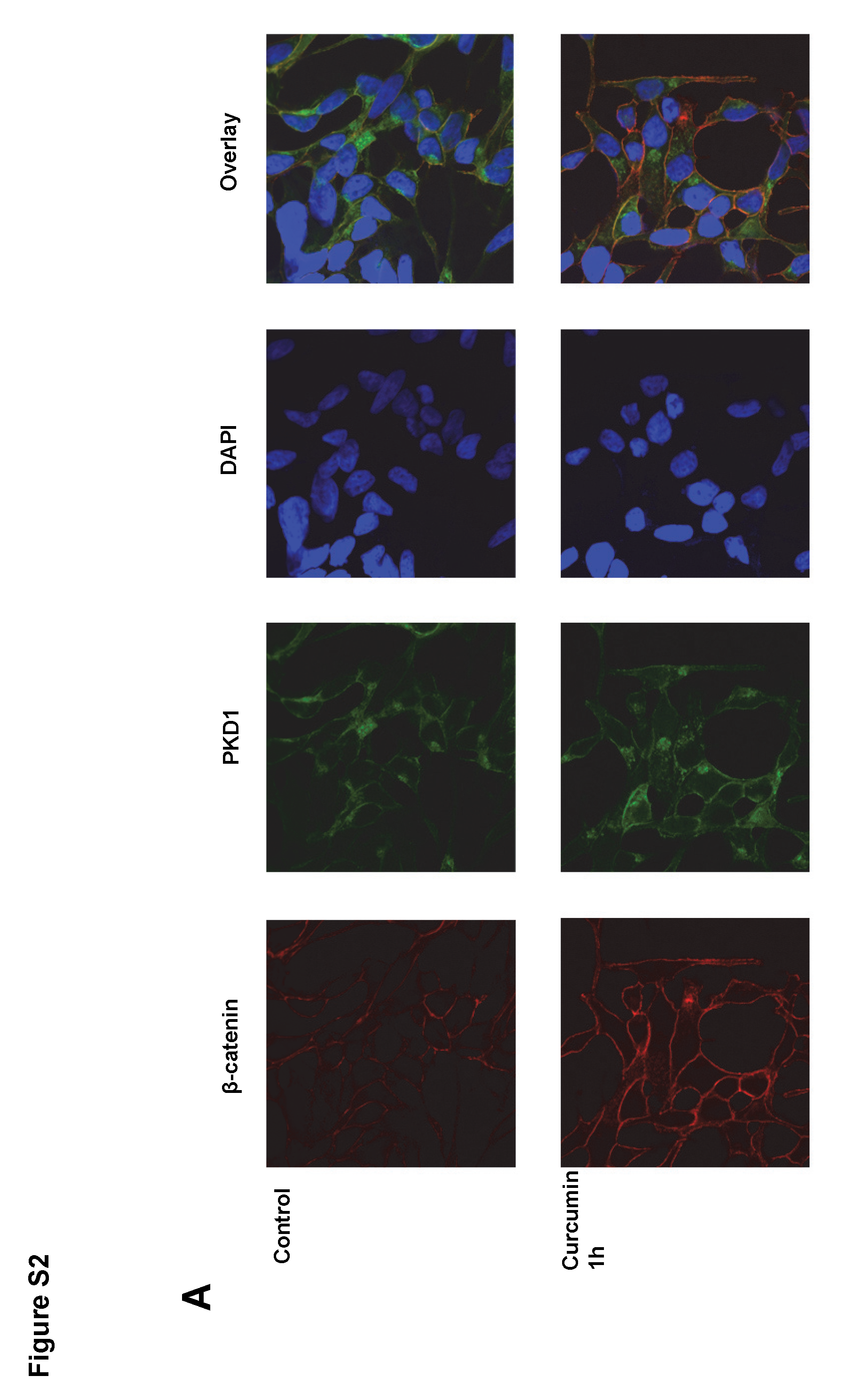

Supplement: Figure S2 — Curcumin treatment enhances membrane β-catenin in LNCaP prostate cancer cells. A). LNCaP cells were cultured on coverslips overnight in 12 well plates and treated with DMSO or curcumin (20 µM) for 1 h. The cells were processed for immunostaining using anti-β-catenin (red) and PKD1 (green) antibodies and counter-stained with DAPI (blue). Enhanced membranous β–catenin staining was observed on cell surface at 1 h of curcumin treatment, compared to control cells treated with vehicle (DMSO). (TIF) [file pone.0035368.s002.tif]

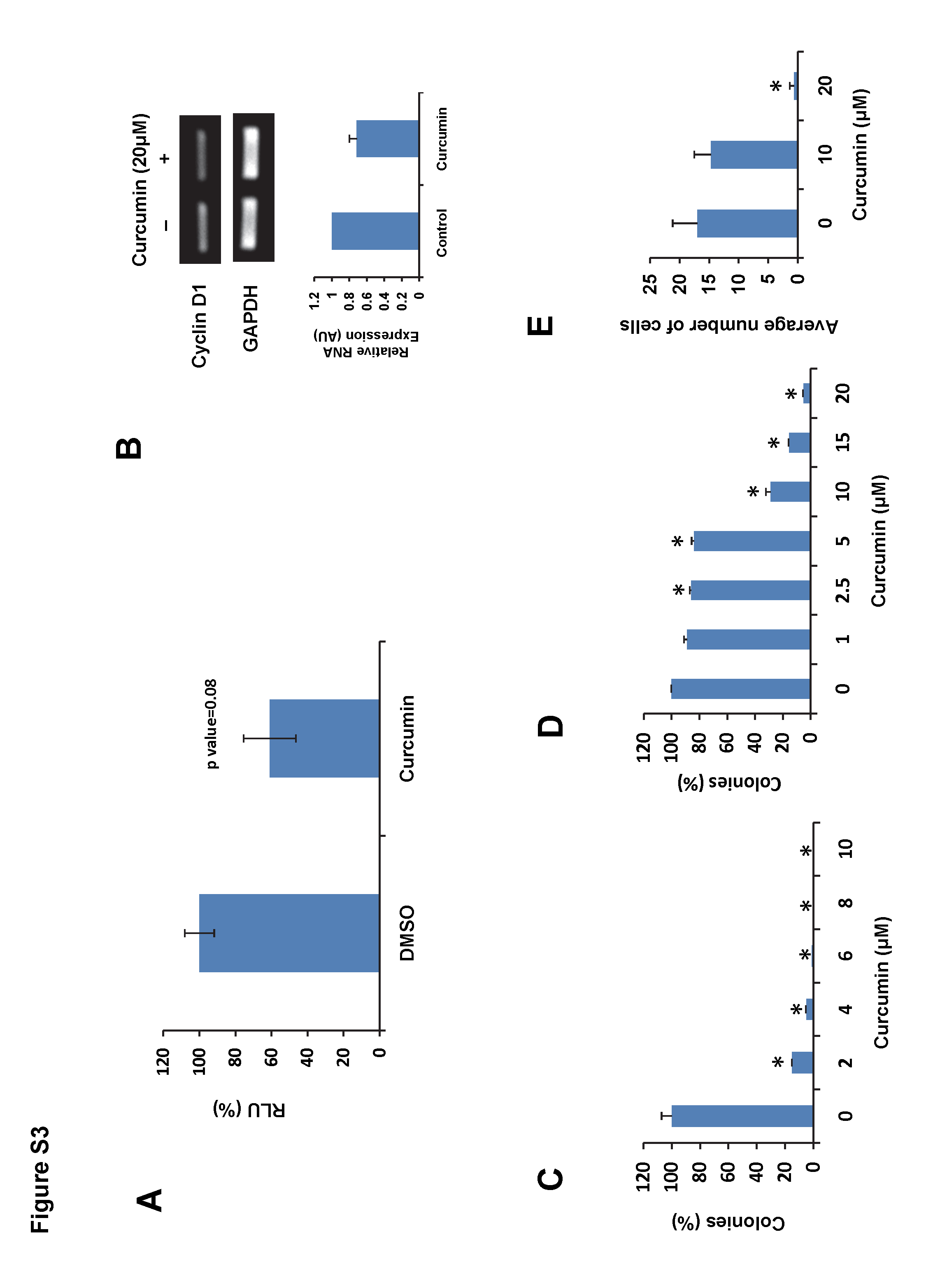

Supplement: Figure S3 — Effect of curcumin treatment on LNCaP prostate cancer cells. A). Effect of curcumin on β-catenin transcription activity in LNCaP prostate cancer cells. A luciferase based reporter assay system was used to measure the β-catenin transcription activity in LNCaP cells, as described in materials and methods and Figure 5. The β-catenin activity of curcumin treated cells was normalized to the activity of vehicle treated cells (considered 100%). Curcumin treatment significantly reduced β-catenin transcription activity in LNCaP cells compared to vehicle treated cells. Mean ± SE, n = 3, *p<0.01. B). Effect of curcumin on cyclin D1 expression in LNCaP cells. RNA was isolated from LNCaP cells treated with curcumin or vehicle control for 24 h and processed for RT-PCR using cyclin D1 and GAPDH specific primers. The densitometric quantitation of cyclin D1 normalized to GAPDH levels is shown in graph. Curcumin treatment specifically reduced the levels of cyclin D1 gene compared to internal control. AU- arbitrary units. C). Anchorage dependent colony formation assay. LNCaP cells (2000) were plated overnight, treated with indicated concentrations of curcumin for 14 days and examined for their colony forming ability. Curcumin showed a dose-dependent inhibition in anchorage dependent colony formation assay. Mean ± SE; n = 3; *p<0.05. D). Anchorage independent colony formation assay. LNCaP cells were seeded in 0.3% agarose and treated with varying concentrations of curcumin for 9 days. The number of colonies were counted and plotted. Curcumin treatment inhibited anchorage independent colony formation of C4-2 cells. Mean ± SE; n = 3; *p<0.01. E) Boyden's chamber assay. Equal numbers of LNCaP cells were seeded on the Boyden's chambers and incubated in the presence DMSO or curcumin for 24 h. Migrated cells were fixed, stained, counted and graphed. Curcumin inhibited motility of LNCaP cells. Mean ± SE; n = 3; *p<0.05. (TIF) [file pone.0035368.s003.tif]

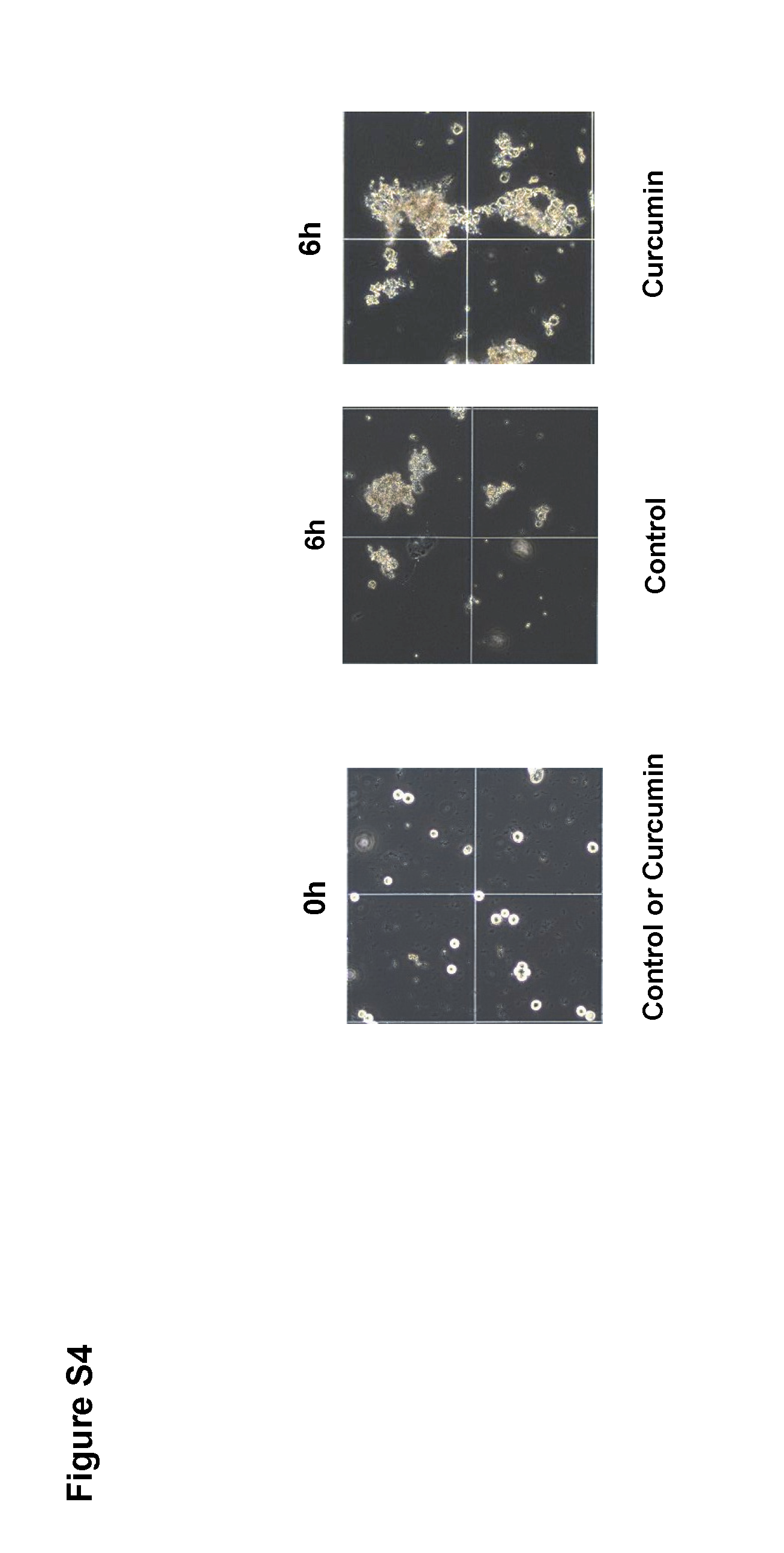

Supplement: Figure S4 — Effect of curcumin treatment on cell-cell aggregation in C4-2-PKD1 cells. C4-2 cells overexpressing PKD1 were treated with curcumin (15 µM) or DMSO for 1 h, harvested and assayed for cell-cell aggregation by incubating under gentle shaking conditions at 37°C in the presence of 5 mM CaCl2. After 6 h incubation, an aliquot of the reaction mixture was photographed for cell-cell aggregation under phase contrast microscope. C4-2-PKD1 cells formed larger cell-cell aggregates than control treatment. Original magnifications 100×. (TIF) [file pone.0035368.s004.tif]
